# Wearable Sensors Reveal Menses-Driven Changes in Physiology and Enable Prediction of the Fertile Window: Observational Study

**DOI:** 10.2196/13404

**Published:** 2019-04-18

**Authors:** Brianna Mae Goodale, Mohaned Shilaih, Lisa Falco, Franziska Dammeier, Györgyi Hamvas, Brigitte Leeners

**Affiliations:** 1 Ava AG Zurich Switzerland; 2 Department of Reproductive Endocrinology University Hospital Zurich Switzerland

**Keywords:** algorithms, fertility/physiology, heart rate, machine learning, menstrual cycle, ovulation detection/methods, respiratory rate, perfusion, skin temperature, wearable electronic devices

## Abstract

**Background:**

Previous research examining physiological changes across the menstrual cycle has considered biological responses to shifting hormones in isolation. Clinical studies, for example, have shown that women’s nightly basal body temperature increases from 0.28 to 0.56 ˚C following postovulation progesterone production. Women’s resting pulse rate, respiratory rate, and heart rate variability (HRV) are similarly elevated in the luteal phase, whereas skin perfusion decreases significantly following the fertile window’s closing. Past research probed only 1 or 2 of these physiological features in a given study, requiring participants to come to a laboratory or hospital clinic multiple times throughout their cycle. Although initially designed for recreational purposes, wearable technology could enable more ambulatory studies of physiological changes across the menstrual cycle. Early research suggests that wearables can detect phase-based shifts in pulse rate and wrist skin temperature (WST). To date, previous work has studied these features separately, with the ability of wearables to accurately pinpoint the fertile window using multiple physiological parameters simultaneously yet unknown.

**Objective:**

In this study, we probed what phase-based differences a wearable bracelet could detect in users’ WST, heart rate, HRV, respiratory rate, and skin perfusion. Drawing on insight from artificial intelligence and machine learning, we then sought to develop an algorithm that could identify the fertile window in real time.

**Methods:**

We conducted a prospective longitudinal study, recruiting 237 conception-seeking Swiss women. Participants wore the Ava bracelet (Ava AG) nightly while sleeping for up to a year or until they became pregnant. In addition to syncing the device to the corresponding smartphone app daily, women also completed an electronic diary about their activities in the past 24 hours. Finally, women took a urinary luteinizing hormone test at several points in a given cycle to determine the close of the fertile window. We assessed phase-based changes in physiological parameters using cross-classified mixed-effects models with random intercepts and random slopes. We then trained a machine learning algorithm to recognize the fertile window.

**Results:**

We have demonstrated that wearable technology can detect significant, concurrent phase-based shifts in WST, heart rate, and respiratory rate (all *P*<.001). HRV and skin perfusion similarly varied across the menstrual cycle (all *P*<.05), although these effects only trended toward significance following a Bonferroni correction to maintain a family-wise alpha level. Our findings were robust to daily, individual, and cycle-level covariates. Furthermore, we developed a machine learning algorithm that can detect the fertile window with 90% accuracy (95% CI 0.89 to 0.92).

**Conclusions:**

Our contributions highlight the impact of artificial intelligence and machine learning’s integration into health care. By monitoring numerous physiological parameters simultaneously, wearable technology uniquely improves upon retrospective methods for fertility awareness and enables the first real-time predictive model of ovulation.

## Introduction

### Background

Wearable sensor technology is evolving rapidly. Primarily providing insights into users’ physical activity, these devices have increasingly been adopted in health care settings [1,2]. Sensors embedded in headbands, chest straps, wristwatches, and clothing itself can now track physiological changes that previously required an electroencephalogram, electrocardiograph, electrodermograph, or electromyograph, respectively [2]. Wearable technology renders medical monitoring accessible to everyday consumers. Recent reviews have noted that these devices may allow for greater longitudinal tracking of physiological parameters, enabling users to see personalized patterns developing in the data. To this end, there remains a dearth of research on the applications of wearable technology in health care, especially in women’s reproductive health.

Wearable sensor technology that helps women track physiological changes across their menstrual cycle could fill the present gap between high-cost, high-accuracy ovulation detection and free, less-precise fertile window approximation. Transvaginal ultrasound examinations represent the gold standard for ovulation detection; however, they are costly and often not feasible in routine clinical settings [3]. Alternatively, women may consider identifying their fertile window using natural family planning (NFP), based on calendar methods [4-6], basal body temperature (BBT) [6,7], and monitoring the amount and consistency of their cervical mucus as it fluctuates in response to changes in estrogen [8,9]. Less technologically sophisticated and thereby less able to pinpoint ovulation exactly, NFP nevertheless helps women recognize physical symptoms that approximate hormonal, phase-driven changes in their body. Fertility tracking may also involve the use of urine-based luteinizing hormone (LH) kits, which detect the LH surge occurring 24 to 36 hours before ovulation [10,11] and are highly correlated with ovulation detected by ultrasonography [12-14].

Although NFP methods enhance fertility awareness, several reviews have highlighted their shortcomings [6,7,15,16]. Most calendar methods, for example, fail to accommodate natural cycle variation, leading to greater inaccuracy [4,5]. NFP practices relying on physical symptoms similarly suffer from methodological pitfalls. Traditional BBT measurements can be influenced by environmental confounds [17] and cannot prospectively predict the fertile window [4,6,18], whereas cervical mucus monitoring relies on subjective patient interpretation of cervical fluid [7]. Finally, urinary LH tests prospectively identify only the last half of the fertile window [7]; women employing this method to achieve pregnancy risk missing the days with the highest probability of conception, which typically occur before a detectable LH surge [4,7]. Critically, NFP methods require sufficient education for correct application, with their success dependent on user motivation and compliance [19].

Advances in mobile phone technology have hinted at the advantages inherent in clinical applications of wearable technology. Smartphone apps designed to facilitate menstrual cycle tracking have simplified and combined NFP methods. Natural Cycles, for example, relies on the calendar method and BBT together to estimate peak fertility [20,21]. Enabling women to track their cycles from home, smartphone apps nevertheless range in accuracy [22]. At best, they can approximate the fertile window using in-app calculations, thereby removing human interpretation and error; however, recent studies citing the incremental improvements of app-based NFP acknowledge their usefulness would be further heightened through biofeedback [23].

Designed to measure and record physiological parameters, wearable technology seems well-suited to address current limitations in traditional and app-based NFP. Their noninvasive nature allows for the convenient, continuous monitoring of multiple parameters simultaneously, resulting in large datasets and individualized pattern tracking via machine learning [2]. For a fraction of the time, cost, and effort, wearables have the potential to reproduce previous findings, demonstrating the correlation between physiological parameters and the menstrual cycle [17,24]. Spontaneously menstruating women show natural variations in body temperature [25], cardiovascular function [26-28], respiratory rate [29,30], and skin perfusion [31,32], depending on their cycle phase. To document these effects, most previous research has required hospital-grade medical equipment (eg, ultrasound machines [33]). Initial research on a wearable fertility tracker, however, has demonstrated that wrist skin temperature (WST) across the menstrual cycle mirrors BBT-measured phase-based changes. Unlike traditional BBT charting, the correlation was robust to potential confounds [17]. A follow-up study demonstrated that heart rate also serves as a reliable, prospective parameter for cycle tracking [24]. Other devices worn on the wrist, under the arm, or in the ear similarly strive to detect menses and the fertile window through monitoring nightly changes in 1 or 2 physiological changes (eg, core body temperature or heart rate [34-37]); to date, peer-reviewed clinical evidence of their efficacy has not yet been published. Wearables have the potential to improve upon digital calendar methods and BBT by measuring multiple physiological parameters continuously, honing in on an even more precise estimation of ovulation [5,6].

### Objectives

Focusing on the application of wearable devices to women’s reproductive health, this study represents the first research to track multiple physiological changes concurrently across the menstrual cycle. Using a clinical sample, we aimed to analyze phase-based differences in skin temperature, heart rate, respiratory rate, perfusion, and heart rate variability (HRV). We also probed the robustness of wearable technology, considering the effect of daily, individual, and cycle-level factors on menstrual phase detection. Finally, drawing on insight from artificial intelligence and machine learning, we sought to develop an algorithm that could identify the 6-day fertile window in real time.

## Methods

### Study Design

Interested in understanding wearable technology’s potential as a fertility aid device, we conducted a correlational prospective cohort study enrolling conception-seeking women. Our dependent variables included nightly physiological readings of each parameter, as predicted by the menstrual cycle phase. In addition, we controlled for participant’s age, body mass index (BMI; kg/m^2^), and other environmental factors that could affect a woman’s heart rate, respiratory rate, HRV, WST, or skin perfusion.

### Participants

In total, 237 women participated in our study. Previous research has demonstrated the difficulty in assessing the necessary sample size to achieve adequate statistical power in multilevel modeling; namely, power analyses require knowing a priori the effect size of interest, each random effect’s variance, covariance estimates for random effects, regression coefficients, and the number of Level 1 groups (eg, how many total cycles and days per cycle each woman will record) [38,39]. As we could not know beforehand the length of each woman’s cycle, we sought to recruit a conservative number of participants based on the sample size used in other comparable clinical studies (eg, ranging from 91 women [17] to 317 women [20]).

We recruited participants via flyers hanging in Zurich-area hospitals and private gynecological offices. In addition, we took out a Facebook advertisement targeting Zurich-area women. Both the Web advertisement and paper flyers directed interested individuals to a website where they were asked to complete an entry questionnaire to evaluate inclusion and exclusion criteria, which were established before the initial study enrollment. To meet eligibility criteria, women had to be aged between 18 and 40 years, have regular menstrual cycles (28 [SD 4] days in length), and be trying to conceive. Individuals who reported doing any hormone therapy currently, had health-related issues that affected their menstrual cycle, were on medications or other substances that could interfere with their menstrual cycle or the physiological parameters investigated, traveled frequently across time zones, had a sleeping disorder, and/or had been trying unsuccessfully to become pregnant for more than a year were excluded from the study. Information on each participant’s weight and height, used to calculate BMI, was also collected.

Eligible individuals were then contacted by the research team and invited to attend an initial meeting at the Department of Reproductive Medicine at the University Hospital, Zurich. A member of the research team met with each person for at least 30 min to verify eligibility and discuss the study protocol. Individuals had up to a week to provide written informed consent, at which time they were equipped with the necessary study materials; informed consent was obtained from all subjects before their study involvement. Participants reported for in-person follow-up appointments after 3, 6, and 9 subsequent menstrual cycles. Each participant remained enrolled in the study for up to a year or until becoming pregnant. Women who returned study materials at the end of their cycle measurements received 120 Swiss francs as compensation for their participation.

### Study Protocol

During their initial meeting with the experimenter, participants received the Web link to a daily diary survey, an electronic wearable to measure physiological parameters while sleeping, and the testing kit for evaluating urinary LH (ClearBlue Advanced Fertility Monitor, SPD Swiss Precision Diagnostics GmbH). Registered with the US Food and Drug Administration as a fertility aid device, the wristworn Ava bracelet (Ava AG) measures 7 physiological parameters simultaneously including WST, heart rate, HRV, respiratory rate, and skin perfusion. The Ava bracelet also measures a user’s sleep quantity and sleep quality. These variables were not of interest to the research question presented here and thus are not included in our analyses. Study participants were instructed to wear the bracelet on the dorsal side of their wrist nightly while sleeping. The electronic wearable automatically saves physiological information every 10 seconds throughout the night. During their initial appointment, participants were shown how to sync the device with the complementary app on their smartphone and were instructed to do so each morning upon waking.

In addition to syncing their bracelet daily, participants also completed a Web diary entry about their activities during the last 24 hours. Previous research has indicated that engaging in aerobic exercise or consuming caffeine, alcohol, or food in the 3 hours before bed can affect physiological parameters of interest to our study (eg, body temperature [40-43]). To control for potential covariates, we asked participants to indicate whether they, in the 3 hours preceding sleep, had sexual intercourse, exercised heavily, eaten, drank coffee, or consumed alcohol. In addition, we asked if participants had taken a pregnancy test that day and, if so, whether they were pregnant.

Finally, participants tracked and reported their LH peak each cycle using the ClearBlue Advanced Fertility Monitor. An at-home LH test, it has been widely used in previous research to estimate the day of ovulation (OV) and close of the fertile window [12,14]. The ClearBlue Fertility Advanced Fertility Monitor shows a smiling face when it detects LH levels indicating *peak fertility* (typically 1 day before OV [14]). From 5 days after the onset of menses and through OV, participants measured their LH levels each morning; they reported their result in their daily diary entry.

In keeping with previous research [17,24], we divided the menstrual cycle into the following 5 phases: (1) *menstruation,* starting with the first day of menses and lasting 5 days; (2) the *follicular phase* **,** starting on the first day post menses and lasting through 6 days before ovulation (OV –6); (3) the *fertile window* **,** starting 5 days before ovulation and lasting through ovulation (OV –5 to OV); (4) the *early luteal phase*, starting 1 day after ovulation through a week after ovulation (OV+1 to OV+7); and (5) the *late luteal phase*, starting 8 days after ovulation (OV+8) and lasting through the day before the onset of menses.

All research was performed in accordance with the Declaration of Helsinki. The clinical protocol was reviewed and approved by the Cantonal Ethics Committee Zurich, Switzerland (BASEC -Nr 2016-02241). It was registered in ClinicalTrials.gov under the identifier NCT03161873 as well as with the Swiss Federal Complementary Database (Portal) before data collection. Informed consent was obtained from all subjects before their study involvement.

### Statistical Analysis

We conducted all data processing and analysis using R (v3.5.1) and Python 3.5. To account for the variation in physiological parameters that arises from sleep onset and awakening [44], we excluded the first 90 and the last 30 min of each night’s data a priori. In addition, each parameter underwent locally estimated scatterplot smoothing (LOESS smoothed) before statistical analysis, thereby reducing artificial fluctuations owing to potential measurement error and consistent with best practices [45]. We tested the underlying assumptions of multilevel modeling by examining the residuals of the 5 base models and plotting their respective q-q plots [46].

Next, to probe whether physiological parameters changed across the menstrual cycle, we ran a series of multilevel models with random slopes and random intercepts. Our data were inherently structured, with nightly measurements nested within menstrual cycles and menstrual cycles nested within participants. Thus, we specified cross-classification in our models. The first random effects term specified participant identification number as the random intercept and the cycle number from which the observation was drawn as the random slope. In the second random effects term, we specified the cycle number as the random intercept and the phases of the menstrual cycle as the random slopes. We optimized the model fit via Residual Maximum Likelihood and Satterthwaite degrees of freedom. Specifically, the R packages (R Foundation for Statistical Computing) lme4 [47], lmerTest [48], optimx [49], and multcomp [50] enabled us to test phase-based changes in physiology across the menstrual cycle. When possible, we chose the model using the percentile of data (stable maxima) with the lowest kurtosis and best fit. When missing data rendered it impossible to compare the fit of 2 models, we conservatively chose the model more closely approximating the median observations (eg, the 70th percentile over the 90th percentile).

Given the large number of covariate models we were testing, we implemented a Bonferroni correction to ensure that the family-wise alpha level did not rise above .05. We divided the desired alpha level of .05 by the total number of models we tested (n=50) to arrive at a revised significance level of less than or equal to .001. We adjusted our definition of marginal significance in turn to comprise an alpha value ranging from .05 (the desired family-wise significance level) to .001. We used the Bonferroni-corrected significance level throughout the paper.

### Creation of the Fertility Algorithm

After retrospectively analyzing the clinical data, we turned to techniques from machine learning to develop an algorithm for predicting and detecting in real time a woman’s fertile window. We used a cycle-based, random 75:25 split for the training and testing datasets with each user belonging to only 1 category; the training dataset consisted of physiological observations from 186 users across 499 cycles, whereas the validation dataset initially contained data from 51 users across 166 cycles. We then trained a random forest with 1000 trees and a max feature parameter of 3 on the training dataset, using the Python module sklearn.ensemble.RandomForestClassifier [51] and the setting max_features=3. We provided 11 input features engineered from the base physiological signals including heart rate, breathing rate, WST, and HRV. We used 3 classes for the initial classification: follicular phase, fertile window, and luteal phase; whereas in our clinical analysis we removed cycles with 20% or more missing data, we kept all cycles in our training dataset. For cycles where participants had synced their data nightly at least 80% of the time, our model used those features in estimating the fertile window. For cycles where nightly data were synced less than 80% of the time, however, the algorithm instead predicted the upcoming fertile window based on the user’s previous cycle length and length of their typical luteal phase.

Following the fertility algorithm’s training, we tested it using the validation dataset to determine its performance. We removed cycles where women had synced less than 80% of the days from the validation dataset before calculating the reported performance metrics, reflecting the manufacturer’s instructions for recommended use. This left us with a validation dataset comprised of 85 cycles spread across 24 users. Interested in our algorithm’s ability to correctly predict the fertile window, we then grouped the follicular and luteal phase classifications together into a single nonfertile comparison group. This reclassification allowed us to calculate the algorithm’s overall accuracy and *F* score. The *F* score serves as a measure of an algorithm’s effectiveness, computed by taking the harmonic average of the mean precision and recall metrics [52].

## Results

### Physiological Changes Across the Menstrual Cycle 

#### Population Characteristics

From the initially recruited 237 participants, we excluded 44 women’s data. In total, 25 participants could not confirm an LH surge in any cycle. In addition, 5 participants had only irregularly long or short menstrual cycles during the course of our study, thereby not meeting the inclusion criteria. Finally, in keeping with previous research and best practices for maximizing fertility prediction algorithms [6,40-43], we also excluded data from 14 women who reported measurements and synchronized their bracelet with the cellphone app less than 80% of days in the cycle.

The final sample included 1194 cycles spread across 193 participants (mean 33.02 [SD 3.68] years); of the 1194 recorded cycles, only 708 met the inclusion criteria for analysis (ie, participants synced their device with the app ≥80% of cycle days and recorded a positive LH test). On average, participants recorded 3.57 analyzable cycles (SD 2.41), with a mean cycle length of 28.21 (SD 2.87) days and a mean BMI of 22.70 kg/m^2^ (SD 3.40). Although some women discontinued study participation after a few cycles to pursue *in vitro* fertilization (n=2) or because they no longer wanted to conceive (n=13), no women cited discomfort from the device as a reason for discontinuation.

### Physiological Changes in Relation to Cycle Phases

**Figure 1:**
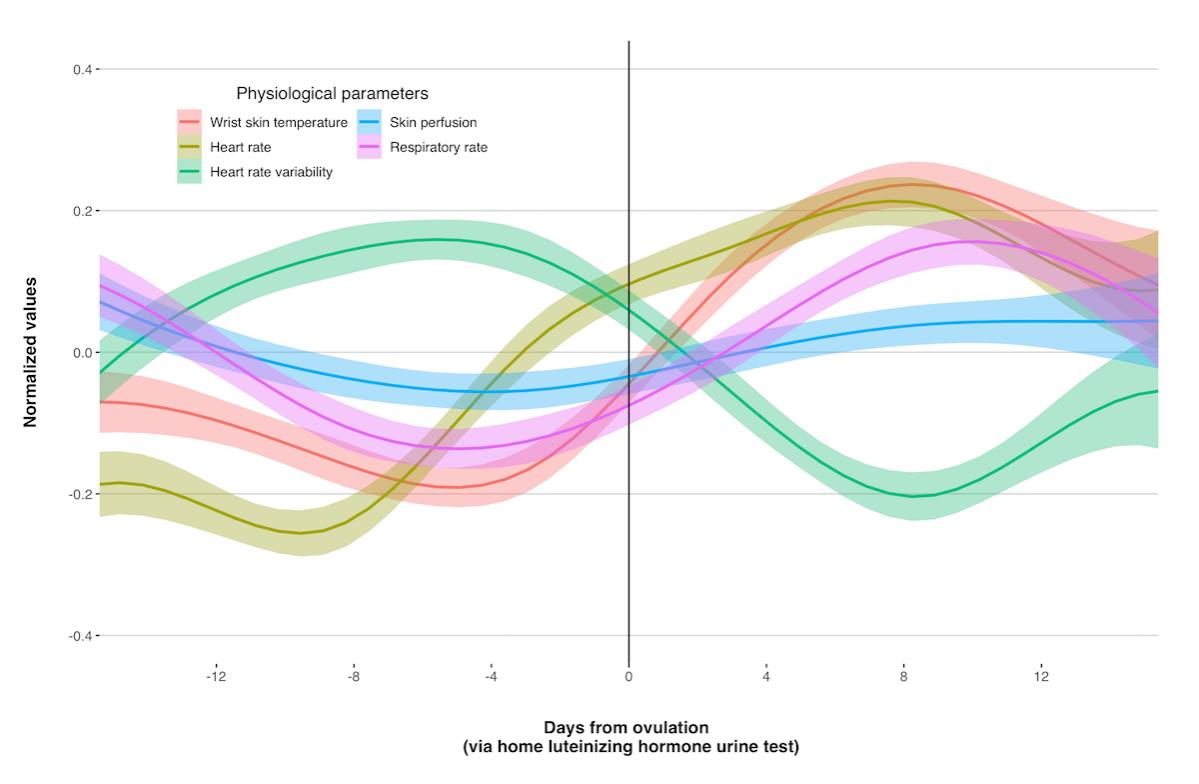
Wearable technology can detect changes in 5 physiological parameters across the menstrual cycle. The smoothed, normalized values of each physiological parameter (with 95% CIs) collapsed across individuals (n=193) and cycles (n=708) are shown, centered around participant-reported luteinizing hormone peak.

We observed significant changes in physiological parameters as captured by the wearable device across the menstrual cycle. Significant findings from the phase-based analysis of each physiological parameter are reported below, with the fixed effects presented in Table 1; the cross-classified full model for each physiological parameter, including random effects, can be found in Multimedia Appendix 1. In addition, changes in physiological parameters across the menstrual cycle are presented concurrently in Figure 1. Across all models, the menstrual phase served as the reference group, with each of the other 4 phases compared with it directly. Furthermore, we tested each base model separately before including potential covariates; unless otherwise noted, individual (eg, BMI and age), cycle-specific (eg, duration), and/or daily (eg, drinking alcohol in the 3 hours preceding sleep) covariates did not change the direction or significance of phase-based effects.

**Table 1:** Multilevel linear mixed models reveal the relationship between menstrual phase and physiological parameters.

Predictors		Wrist skin temperature^a^	Heart rate^a^	Heart rate variability^a^	Respiratory rate^a^	Skin perfusion^a^
Intercept		34.08^b^ (0.08)	58.62^b^ (0.44)	1.70^b^ (0.10)	16.92^b^ (0.14)	1306.54^b^ (75.39)
**Cycle phase**						
	Menstrual	Reference group	Reference group	Reference group	Reference group	Reference group
	Follicular	−0.24^b^ (0.02)	−1.54^b^ (0.20)	0.11^c^ (0.03)	−0.39^b^ (0.04)	−44.32^c^ (14.80)
	Fertile	−0.25^b^ (0.03)	−0.03 (0.26)	0.08^c^ (0.03)	−0.48^b^ (0.04)	−73.58^c^ (18.25)
	Early luteal	0.01 (0.02)	2.01^b^ (0.20)	−0.11 (0.05)	−0.20^b^ (0.04)	−12.02 (18.49)
	Late luteal	0.20^b^ (0.02)	2.46^b^ (0.29)	−0.20^c^ (0.04)	0.22^b^ (0.03)	51.33 (26.96)

^a^Unstandardized b-coefficient values reported, with SEs in parentheses.

^b^*P*<.001 with a Bonferroni correction.

^c^*P*<.05 with a Bonferroni correction.

#### Wrist Skin Temperature

Regressing nightly WST on menstrual phases using the 50th percentile data revealed women had a significantly lower WST in the follicular phase (mean 33.87 °C [SD 0.84]; *t*_5.06_=–11.12; *P<*.001) and fertile window (mean 33.88 °C [SD 0.78]; *t*_4.96_=–8.97; *P<*.001) compared with menses (mean 34.11 °C [SD 0.84]). WST was also significantly higher in the late luteal (mean 34.32 °C [SD 0.82];*t*_14.80_=10.96; *P<*.001) phase compared with menses.

Controlling for individual, cycle-level, or daily covariates did not change the effect of menstrual phase on WST. In general, women with higher BMIs had significantly lower nightly WST (*t*_184.69_=–3.70; *P* *<*.001). Compared with nights when she did not eat or had only a small meal in the 3 hours before sleep, a woman had significantly lower WST after eating a medium- (*t*_16220_=–3.58; *P* *<*.001) or large-sized meal (*t*_16240_=–5.10; *P<*.001; see Table 2).

#### Heart Rate

There was a significant effect of cycle phase on average nightly heart rate in the data drawn from the 30th percentile. In particular, heart rate was significantly lower in the follicular phase (mean 56.56 [SD 6.29] beats per minute [bpm]) compared with the menstrual phase (mean 57.92 [SD 6.27] bpm; *t*_8.43_=–7.66; *P<*.001). Nightly heart rate was significantly higher in the early (mean 59.98 [SD 6.50] bpm; *t*_5.74_=9.93; *P<*.001) and late luteal phases (mean 60.47 [SD 6.45] bpm; *t*_6.02_=8.53; *P<*.001) than in menses, however.

Daily and cycle-level variables significantly affected nightly heart rate, over and above the effect of the menstrual phase. Women who ate a large-sized meal (*t*_16220_=8.16; *P<*.001), drank at least 1 serving of alcohol (1 to 4 units, *t*_16250_=12.43; *P<*.001; ≥5 units, *t*_16250_=18.28; *P<*.001), and/or exercised for at least 60 min (*t*_16280_=4.84; *P<*.001) in the 3 hours before sleep had significantly higher heart rates on a given night. Finally, during a longer cycle, women were significantly more likely to have an increased heart rate on a given night (*t*_14000_=5.44; *P<*.001). Inclusion of these covariates in the model did not affect the direction or significance of phase-based effects (see Table 3).

**Table 2:** Multilevel linear mixed models reveal the relationship between menstrual phase, covariates, and wrist skin temperature.

Predictors		Model 1^a^	Model 2^a^	Model 3^a^	Model 4^a^	Model 5^a^	Model 6^a^	Model 7^a^	Model 8^a^	Model 9^a^
Intercept		34.12^b^ (0.08)	35.40^b^ (0.36)	34.09^b^ (0.08)	34.09^b^ (0.08)	34.09^b^ (0.08)	34.09^b^ (0.08)	33.32^b^ (0.48)	34.24^b^ (0.10)	35.01^b^ (0.64)
**Cycle phase**										
	Menstrual	Reference group	Reference group	Reference group	Reference group	Reference group	Reference group	Reference group	Reference group	Reference group
	Follicular	–0.24^b^ (0.03)	–0.23^b^ (0.03)	–0.24^b^ (0.03)	–0.24^b^ (0.03)	–0.24^b^ (0.02)	–0.24^b^ (0.03)	–0.24^b^ (0.03)	–0.24^b^ (0.03)	–0.23^b^ (0.03)
	Fertile	–0.26^b^ (0.03)	–0.25^b^ (0.03)	–0.26^b^ (0.03)	–0.26^b^ (0.03)	–0.25^b^ (0.03)	–0.26^b^ (0.03)	–0.26^b^ (0.03)	–0.26^b^ (0.03)	–0.24^b^ (0.03)
	Early luteal	0.00 (0.02)	0.01 (0.03)	0.00 (0.02)	0.00 (0.02)	0.01 (0.02)	0.00 (0.02)	0.00 (0.03)	0.00 (0.02)	0.02 (0.03)
	Late luteal	0.19^b^ (0.02)	0.20^b^ (0.02)	0.19^b^ (0.02)	0.19^b^ (0.02)	0.19^b^ (0.02)	0.19^b^ (0.02)	0.20^b^ (0.02)	0.19^b^ (0.02)	0.20^b^ (0.02)
**Meal^c^**										
	Small or no food	Reference group	—^d^	—	—	—	—	—	—	Reference group
	Medium meal	–0.03^b^ (0.01)	—	—	—	—	—	—	—	–0.03^b^ (0.01)
	Large meal	–0.05^b^ (0.01)	—	—	—	—	—	—	—	–0.05^b^ (0.01)
Body mass index (kg/m^2^)		—	–0.06^b^ (0.02)	—	—	—	—	—	—	–0.05^b^ (0.02)
Coffee^c^		—	—	0.00 (0.01)	—	—	—	—	—	0.01 (0.01)
**Exercise^c^**										
	No exercise	—	—	—	Reference group	—	—	—	—	Reference group
	<60 min	—	—	—	0.01 (0.01)	—	—	—	—	0.01 (0.01)
	>60 min	—	—	—	0.02 (0.01)	—	—	—	—	0.01 (0.01)
Sexual intercourse^c^		—	—	—	—	–0.02^e^ (0.01)	—	—	—	–0.02^e^ (0.01)
**Alcohol^c^**										
	No alcohol	—	—	—	—	—	Reference group	—	—	Reference group
	1-4 units	—	—	—	—	—	–0.01 (0.01)	—	—	0.00 (0.01)
	≥5 units	—	—	—	—	—	0.05^e^ (0.02)	—	—	0.07^d^ (0.02)
Age (years)		—	—	—	—	—	—	0.02 (0.01)	—	0.02 (0.01)
Cycle duration		—	—	—	—	—	—	—	–0.01^e^ (0.00)	–0.01^e^ (0.00)

^a^Unstandardized b-coefficient values reported, with SEs in parentheses.

^b^*P*<.001 with a Bonferroni correction.

^c^Within the 3 hours preceding the onset of sleep.

^d^Indicates a predictor was not considered in a given model.

^e^*P*<.05 with a Bonferroni correction.

**Table 3:** Multilevel linear mixed models reveal the relationship between menstrual phase, covariates, and heart rate.

Predictors		Model 1^a^	Model 2^a^	Model 3^a,b^	Model 4^a,b^	Model 5^a^	Model 6^a^	Model 7^a^	Model 8^a,b^	Model 9^a^
Intercept		58.40^c^ (0.44)	54.02^c^ (2.86)	58.58^c^ (0.44)	58.53^c^ (0.44)	58.60^c^ (0.44)	58.38^c^ (0.44)	60.37^c^ (3.81)	56.51^c^ (0.58)	53.52^c^ (5.06)
**Cycle phase**										
	Menstrual	Reference group	Reference group	Reference group	Reference group	Reference group	Reference group	Reference group	Reference group	Reference group
	Follicular	–1.57^c^ (0.21)	–1.57^c^ (0.21)	–1.29^c^ (0.07)	–1.30^c^ (0.07)	1.57^c^ (0.21)	–1.58^c^ (0.22)	–1.55^c^ (0.20)	–1.31^c^ (0.07)	–1.62^c^ (0.22)
	Fertile	–0.04 (0.25)	–0.03 (0.23)	0.19^d^ (0.07)	0.19^d^ (0.07)	–0.04 (0.25)	–0.04 (0.24)	–0.02 (0.24)	0.19^d^ (0.06)	–0.02 (0.24)
	Early luteal	1.95^c^ (0.19)	1.98^c^ (0.16)	2.01^c^ (0.06)	2.01^c^ (0.06)	1.95^c^ (0.18)	1.98^c^ (0.18)	1.96^c^ (0.17)	2.01^c^ (0.06)	2.00^c^ (0.17)
	Late luteal	2.39^c^ (0.24)	2.40^c^ (0.24)	2.37^c^ (0.07)	2.37^c^ (0.07)	2.40^c^ (0.24)	2.41^c^ (0.25)	2.40^c^ (0.24)	2.37^c^ (0.07)	2.40^c^ (0.25)
**Meal^d^**										
	Small or no food	Reference group	—^e^	—	—	—	—	—	—	Reference group
	Medium meal	0.08 (0.06)	—	—	—	—	—	—	—	0.05 (0.06)
	Large meal	0.56^c^ (0.07)	—	—	—	—	—	—	—	0.31^c^ (0.07)
Body mass index (kg/m^2^)		—	0.21 (0.12)	—	—	—	—	—	—	0.20 (0.13)
Coffee^d^		—	—	0.02 (0.08)	—	—	—	—	—	–0.14 (0.08)
**Exercise^d^**										
	No exercise	—	—	—	Reference group	—	—	—	—	Reference group
	<60 min	—	—	—	0.03 (0.06)	—	—	—	—	0.05 (0.06)
	>60 min	—	—	—	0.33^c^ (0.07)	—	—	—	—	0.36^c^ (0.07)
Sexual intercourse^d^		—	—	—	—	0.03 (0.06)	—	—	—	–0.04 (0.06)
**Alcohol^d^**										
	No alcohol	—	—	—	—	—	Reference group	—	—	Reference group
	1-4 units	—	—	—	—	—	0.62^c^ (0.05)	—	—	0.53^c^ (0.05)
	≥5 units	—	—	—	—	—	2.82^c^ (0.15)	—	—	2.70^c^ (0.16)
Age (years)		—	—	—	—	—	—	–0.05 (0.11)	—	–0.05 (0.12)
Cycle duration		—	—	—	—	—	—		0.07^c^ (0.01)	0.06^c^ (0.01)

^a^Unstandardized b-coefficient values reported, with SEs in parentheses.

^b^The model would not converge with the cross-classification term, so only random intercepts were included.

^c^*P*<.001 with a Bonferroni correction.

^d^*P*<.05 with a Bonferroni correction.

^d^Within the 3 hours preceding the onset of sleep.

^e^Indicates a predictor was not considered in a given model.

#### Heart Rate Variability

Analysis of data from the 90th percentile revealed a marginally significant effect of cycle phase on the criterion. The average nightly HRV ratio was higher in the follicular phase (mean 1.86 [SD 0.91]; *t*_4.10_=3.28; *P=*.03) and fertile window (mean 1.86 [SD 0.91]; *t*_3.49_=3.37; *P=*.03) than in the menstrual phase (mean 1.78 [SD 0.88]). The HRV ratio dipped during the luteal phase, tending to be lower on a given night in the late luteal phase (mean 1.62 [SD 0.79]) than during menses (*t*_4.38_=–5.50; *P=*.004). Not meeting the more conservative Bonferroni corrected alpha level of .001, however, these phase-based differences only trended toward significance.

A covariate analysis revealed only a significant effect of daily-level predictors on HRV, over and above the effects of the menstrual phase. In particular, women had lower HRV ratios on a given night if they had eaten a large-sized meal (*t*_16280_=–4.35; *P<*.001) compared with nights where they fasted or ate only a small meal in the 3 hours before bed. Nevertheless, the phase-based trends in HRV ratio remained robust; compared with menses, the HRV ratio was higher in the follicular phase and fertile window, but lower during the luteal phase (see Table 4).

#### Respiratory Rate

Examining data from the 90th percentile of nightly observations, respiratory rate was significantly lower in the follicular phase (mean 16.57 [SD 2.06] breaths/min; *t*_8.55_=–9.96; *P<*.001), fertile window (mean 16.40 [SD 2.00] breaths/min; *t*_9.52_=–11.59; *P<*.001), and early luteal phase (mean 16.68 [SD 1.96] breaths/min; *t*_9.39_=–5.44; *P<*.001) compared with menses (mean 16.86 [SD 2.03] breaths/min). Finally, during the late luteal phase, a woman’s respiratory rate was significantly faster (mean 17.04 [SD 1.97] breaths/min; *t*_7.52_=6.74; *P<*.001) than during menses.

Eating a large meal (*t*_16210_=5.39; *P<*.001) or consuming alcohol (1 to 4 units, *t*_16200_=8.86; *P<*.001; ≥5 units, *t*_16210_=12.56; *P<*.001) in the 3 hours preceding sleep was associated with a significant increase in nightly respiratory rate, over and above the effects of the menstrual phase. When considered alongside all measured covariates in a single model, having sexual intercourse significantly decreased a woman’s nightly respiratory rate compared with nights where she was abstinent, over and above the effects of the menstrual phase (*t*_15250_=–3.50; *P*<.001). Accounting for the effects of individual covariates, however, did not alter the direction or significance of the menstrual phase on respiratory rate (see Table 5).

#### Skin Perfusion

Mixed-effects modeling with random slopes and random intercepts using the 90th percentile data revealed a marginally significant effect of cycle phase on skin perfusion; on average, wrist skin was less perfuse during the follicular phase (mean 1438.78 [SD 599.43]; *t*_6.33_=–3.00; *P=*.02) and the fertile window (mean 1384.02 [SD 577.19]; *t*_7.37_=–4.03; *P=*.004) compared with menses (mean 1431.53 [SD 592.22]).

Inclusion of covariates in the base model did not affect the direction of the relationship between menses and the follicular phase or menses and the fertile window (see Table 6). Drinking 1 to 4 units of alcohol (*t*_16247.87_=–4.11; *P<*.001) in the 3 hours before sleep significantly decreased nightly skin perfusion, over and above the marginally significant effects of cycle phase. In addition, eating a medium- (*t*_16237.84_=–3.67; *P<*.001) or large-sized meal (*t*_16254.02_=–5.85; *P<*.001) in the 3 hours preceding sleep significantly reduced nightly perfusion, compared with when a woman fasted or had only a small meal before bed.

**Table 4:** Multilevel linear mixed models reveal the relationship between the menstrual phase, covariates, and heart rate variability.

Predictors		Model 1^a^	Model 2^a^	Model 3^a^	Model 4^a,b^	Model 5^a,b^	Model 6^a,b^	Model 7^a^	Model 8^a^	Model 9^a,b^
Intercept		1.75^c^ (0.10)	1.51^c^ (0.37)	1.73^c^ (0.10)	1.74^c^ (0.09)	1.74^c^ (0.09)	1.75^c^ (0.09)	0.77 (0.49)	1.70^c^ (0.11)	0.32 (0.64)
**Cycle phase**										
	Menstrual	Reference group	Reference group	Reference group	Reference group	Reference group	Reference group	Reference group	Reference group	Reference group
	Follicular	0.12^d^ (0.03)	0.12^d^ (0.03)	0.12^d^ (0.03)	0.11^c^ (0.01)	0.11^c^ (0.01)	0.11^c^ (0.01)	0.12^d^ (0.03)	0.17^d^ (0.03)	0.11^c^ (0.01)
	Fertile	0.08^d^ (0.02)	0.07^d^ (0.02)	0.08^d^ (0.02)	0.07^c^ (0.01)	0.07^c^ (0.01)	0.07^c^ (0.01)	0.07^d^ (0.02)	0.08^d^ (0.02)	0.06^c^ (0.01)
	Early luteal	–0.12^d^ (0.04)	–0.11^d^ (0.04)	–0.11^d^ (0.04)	–0.11^c^ (0.01)	–0.11^c^ (0.01)	–0.11^c^ (0.01)	–0.12^d^ (0.04)	–0.12^d^ (0.04)	–0.11^c^ (0.01)
	Late luteal	–0.20^d^ (0.04)	–0.20^d^ (0.04)	–0.12^d^ (0.01)	–0.19^c^ (0.01)	–0.19^c^ (0.01)	–0.19^c^ (0.01)	–0.20^d^ (0.04)	–0.20^d^ (0.04)	–0.19^c^ (0.01)
**Meal^e^**										
	Small or no food	Reference group	—^f^	—	—	—	—	—	—	Reference group
	Medium meal	–0.02^d^ (0.01)	—	—	—	—	—	—	—	–0.02^e^ (0.01)
	Large meal	–0.04^c^ (0.01)	—	—	—	—	—	—	—	–0.03^e^ (0.01)
Body mass index (kg/m^2^)		—	0.01 (0.02)	—	—	—	—	—	—	0.01 (0.02)
Coffee^e^		—	—	–0.03^d^ (0.01)	—	—	—	—	—	–0.02 (0.01)
**Exercise^e^**										
	No exercise	—	—	—	Reference group	—	—	—	—	Reference group
	<60 min	—	—	—	0.01 (0.01)	—	—	—	—	0.01 (0.01)
	>60 min	—	—	—	0.01 (0.01)	—	—	—	—	0.00 (0.01)
Sexual intercourse^e^		—	—	—	—	–0.02 (0.01)	—	—	—	–0.01 (0.01)
**Alcohol^e^**										
	No alcohol	—	—	—	—	—	Reference group	—	—	Reference group
	1-4 units	—	—	—	—	—	–0.02^d^ (0.01)	—	—	–0.01(0.01)
	≥5 units	—	—	—	—	—	–0.06^d^ (0.02)	—	—	–0.05(0.02)
Age (years)		—	—	—	—	—	—	0.03^d^ (0.01)	—	0.03^d^ (0.01)
Cycle duration		—	—	—	—	—	—	—	0.00 (0.00)	0.00 (0.00)

^a^Unstandardized b-coefficient values reported, with SEs in parentheses.

^b^The model would not converge with the cross-classification term, so only random intercepts were included.

^c^*P*<.001 with a Bonferroni correction.

^d^*P*<.05 with a Bonferroni correction.

^e^Within the 3 hours preceding the onset of sleep.

^f^Indicates a predictor was not considered in a given model.

**Table 5:** Multilevel linear mixed models reveal the relationship between menstrual phase, covariates, and respiratory rate.

Predictors		Model 1^a^	Model 2^a^	Model 3^a^	Model 4^a^	Model 5^a^	Model 6^a^	Model 7^a^	Model 8^a^	Model 9^a^
Intercept		16.89^b^ (0.14)	15.39^b^ (0.97)	16.91^b^ (0.14)	16.92^b^ (0.14)	16.92^b^ (0.14)	16.88^b^ (0.14)	17.82^b^ (1.28)	16.88^b^ (0.17)	16.15^b^ (1.72)
**Cycle phase**										
	Menstrual	Reference group	Reference group	Reference group	Reference group	Reference group	Reference group	Reference group	Reference group	Reference group
	Follicular	–0.38^b^ (0.04)	–0.39^b^ (0.05)	–0.38^b^ (0.04)	–0.38^b^ (0.04)	–0.38^b^ (0.04)	–0.38^b^ (0.05)	–0.38^b^ (0.05)	–0.38^b^ (0.04)	–0.39^b^ (0.05)
	Fertile	–0.48^b^ (0.04)	–0.48^b^ (0.04)	–0.47^b^ (0.04)	–0.48^b^ (0.04)	–0.46^b^ (0.04)	–0.48^b^ (0.04)	–0.47^b^ (0.04)	–0.47^b^ (0.04)	–0.46^b^ (0.04)
	Early luteal	–0.21^b^ (0.04)	–0.22^b^ (0.04)	–0.21^b^ (0.04)	–0.21^b^ (0.04)	–0.21^b^ (0.04)	–0.21^b^ (0.04)	–0.21^b^ (0.04)	–0.21^b^ (0.04)	–0.21^b^ (0.04)
	Late luteal	0.21^b^ (0.03)	0.21^b^ (0.03)	0.22^b^ (0.03)	0.22^b^ (0.03)	0.22^b^ (0.03)	0.21^b^ (0.03)	0.22^b^ (0.03)	0.22^b^ (0.03)	0.22^b^ (0.03)
**Meal^c^**										
	Small or no food	Reference group	—^d^	—	—	—	—	—	—	Reference group
	Medium meal	0.00 (0.02)	—	—	—	—	—	—	—	–0.01 (0.02)
	Large meal	0.09^b^ (0.02)	—	—	—	—	—	—	—	0.04^e^ (0.02)
Body mass index (kg/m^2^)		—	0.07 (0.04)	—	—	—	—	—	—	0.06 (0.04)
Coffee^c^		—	—	.06^e^ (0.02)	—	—	—	—	—	0.03 (0.02)
**Exercise^c^**										
	No exercise	—	—	—	Reference group	—	—	—	—	Reference group
	<60 min	—	—	—	–0.01 (0.02)	—	—	—	—	–0.01 (0.02)
	>60 min	—	—	—	0.03 (0.02)	—	—	—	—	0.03 (0.02)
Sexual intercourse^c^		—	—	—	—	–0.04^e^ (0.01)	—	—	—	–0.05^b^ (0.02)
**Alcohol^c^**										
	No alcohol	—	—	—	—	—	Reference group	—	—	Reference group
	1-4 units	—	—	—	—	—	0.11^b^ (0.01)	—	—	0.10^b^ (0.01)
	>5 units	—	—	—	—	—	0.48^b^ (0.04)	—	—	0.46^b^ (0.04)
Age (years)		—	—	—	—	—	—	–0.03 (0.04)	—	–0.02 (0.04)
Cycle duration		—	—	—	—	—	—	—	0.00 (0.00)	0.00 (0.00)

^a^Unstandardized b-coefficient values reported, with SEs in parentheses.

^b^*P*<.001 with a Bonferroni correction.

^c^Within the 3 hours preceding the onset of sleep.

^d^Indicates a predictor was not considered in a given model.

^e^*P*<.05 with a Bonferroni correction.

**Table 6:** Multilevel linear mixed models reveal the relationship between menstrual phase, covariates, and skin perfusion.

Predictors		Model 1^a^	Model 2^a,b^	Model 3^a^	Model 4^a,b^	Model 5^a^	Model 6^a^	Model 7^a,b^	Model 8^a^	Model 9^a,b^
Intercept		1338.17^c^ (72.65)	811.67^d^ (244.86)	1308.46^c^ (72.58)	1316.50^c^ (74.72)	1308.46^c^ (72.59)	1315.27^c^ (72.86)	1629.12^c^ (319.49)	1325.16^c^ (85.35)	1080.00^d^ (426.30)
**Cycle phase**										
	Menstrual	Reference group	Reference group	Reference group	Reference group	Reference group	Reference group	Reference group	Reference group	Reference group
	Follicular	–49.62^d^ (15.07)	–27.30^c^ (8.28)	–49.94^d^ (15.20)	–30.93^c^ (8.27)	–48.22^d^ (15.12)	–50.23^d^ (15.28)	–30.89^c^ (8.32)	–49.29^d^ (15.09)	–26.76^d^ (8.35)
	Fertile	–68.23^d^ (16.94)	–45.20^c^ (7.69)	–68.77^d^ (17.03)	–48.69^c^ (7.70)	–65.00^d^ (16.99)	–68.17^d^ (16.74)	–48.91^c^ (7.74)	–68.04^d^ (16.87)	–43.89^c^ (7.96)
	Early luteal	–6.37 (17.48)	–15.96^d^ (7.50)	–7.14 (17.37)	–12.76 (7.51)	–5.64 (17.54)	–6.88 (17.56)	–14.68 (7.54)	–7.06 (17.68)	–1.53^d^ (7.53)
	Late luteal	60.82 (28.62)	34.01^c^ (8.09)	60.73 (28.60)	33.85^c^ (8.11)	61.94 (28.71)	60.18 (28.75)	33.83^c^ (8.14)	61.94 (29.06)	33.43^c^ (8.13)
**Meal^e^**										
	Small or no food	Reference group	—^f^	—	—	—	—	—	—	Reference group
	Medium meal	–26.43^c^ (7.21)	—	—	—	—	—	—	—	–20.55^d^ (7.29)
	Large meal	–47.36^c^ (8.09)	—	—	—	—	—	—	—	–40.31^c^ (8.52)
Body mass index (kg/m^2^)		—	22.08^d^ (10.21)	—	—	—	—	—	—	21.19^d^ (10.33)
Coffee^e^		—	—	–7.00 (9.77)	—	—	—	—	—	–0.04 (9.77)
**Exercise^e^**										
	No exercise	—	—	—	Reference group	—	—	—	—	Reference group
	<60 min	—	—	—	–17.81^d^ (7.46)	—	—	—	—	–18.56^d^ (7.45)
	>60 min	—	—	—	11.12 (8.03)	—	—	—	—	7.65 (8.11)
Sexual intercourse^e^		—	—	—	—	–11.42 (7.00)	—	—	—	–4.90 (7.03)
**Alcohol^e^**										
	No alcohol	—	—	—	—	—	Reference group	—	—	Reference group
	1-4 units	—	—	—	—	—	–24.71^c^ (6.01)	—	—	–16.89^d^ (6.33)
	>5 units	—	—	—	—	—	–17.64 (18.50)	—	—	–13.31 (18.56)
Age (years)		—	—	—	—	—	—	–9.56 (9.36)	—	–6.59 (9.49)
Cycle duration		—	—	—	—	—	—	—	–0.62 (1.59)	–0.01 (1.61)

^a^Unstandardized b-coefficient values reported, with SEs in parentheses.

^b^The model would not converge with the cross-classification term, so only random intercepts were included.

^c^*P*<.001 with a Bonferroni correction.

^d^*P*<.05 with a Bonferroni correction.

^e^Within the 3 hours preceding the onset of sleep.

^f^Indicates a predictor was not considered in a given model.

#### Fertility Prediction Algorithm Performance

Having demonstrated the significant changes in physiological parameters across the menstrual cycle, we proceeded to develop a predictive real-time model for detection of the fertile window. Employing an ensemble tree-based machine learning method resulted in good separation between the different phases of the menstrual cycle, with some phases easier to isolate than others (see Multimedia Appendix 2 for the confusion matrix). The overall method performance based on the *F* score was 0.78 (95% CI 0.74 to 0.82; specificity=0.93, 95% CI 0.92 to 0.94; sensitivity=0.81, 95% CI 0.77 to 0.85); furthermore, the algorithm accurately detected the 6-day fertile window in 90% of cycles (95% CI 0.89 to 0.92; see Multimedia Appendix 3 for full performance metrics). A 2018 review of smartphone apps for tracking the menstrual cycle found that fewer than a third of the 73 apps surveyed (17.3%) could predict a 6-day or smaller fertile window, achieving an accuracy between 11% and 81% (mean 53% [SD 21%]) [53]. In addition to having a shorter fertile window than most of the apps studied, our algorithm had a higher accuracy metric. Although NFP techniques have demonstrated an achieved accuracy of up to 98%, they require assumptions about the next cycle’s duration to determine accuracy and do so at the expense of providing a broader fertile window (ranging from 8 to 64 days for the rhythm method) [53]. By drawing on machine learning and users’ detailed physiological profiles across multiple cycles, our fertility prediction algorithm achieves higher accuracy than previous smartphone apps and pinpoints a fertile window more precisely than traditional NFP.

## Discussion

### Principal Findings

Probing wearable technology’s ability to monitor multiple physiological parameters concurrently, our study demonstrated how WST, heart rate, HRV, respiratory rate, and skin perfusion vary across the menstrual cycle. In line with previous research [17], we captured a biphasic shift in WST; compared with menses, women had significantly lower WST in the follicular and fertile phase and significantly higher WST in the late luteal phase. The phase-based changes in WST mirror findings from studies using more traditional BBT methods for temperature tracking [4,6,31]. Wearable technology renders similar readings and conclusions as BBT in a less invasive manner, solving many of BBT’s inherent disadvantages (eg, the need to take one’s temperature at the same time daily [7]). Our study also upholds conclusions from previous research, which revealed women have significantly higher resting pulse rates [26,28-30,54] and respiratory rates [29,30] during the luteal phase compared with earlier in their menstrual cycle. Although we have previously captured phase-based changes for a single parameter in an ambulatory setting [17,24], we have demonstrated here for the first time that wearable technology can track multiple physiological parameters across the menstrual cycle simultaneously. In turn, this increase in recorded features allowed us to harness machine learning to predict the opening and closing of the fertile window with high accuracy.

### Limitations

Not all phase-based changes in physiological parameters manifested as we predicted, however. Previous research considering the effect of the menstrual cycle on perfusion led us to expect significantly less skin perfusion in the luteal and menstrual phases compared with the follicular phase or fertile window [55]. However, our results trended in the opposite direction, with skin perfusion lower during the follicular phase and fertile window compared with menses. Methodological differences in protocol may partially explain the discrepancy between our findings and past work. Although most researchers measured skin perfusion via sensors on a participant’s finger or their forearm [55], participants in this study wore our device on the dorsal side of their wrist. Previous research has found that, even within the same study, population, and time frame, conclusions about the physiological changes in skin perfusion across the menstrual cycle may differ depending on where on a participant’s finger the measuring instrument was placed [56]. Future research should consider how the location of sensors monitoring peripheral blood flow moderates phase-based differences in skin perfusion.

Like skin perfusion, HRV also showed marginally significant phase-based changes across the menstrual cycle in the opposite direction than anticipated. Multiple studies have reported women have higher HRV ratios during the luteal phase than earlier in their cycle [27,28,54]. In contrast, we found that the HRV ratio increased during the follicular phase compared with menses before decreasing during the luteal phase. Although a definitive explanation would require follow-up studies, we believe this difference may be due to variability in the experimental context. Owing to wearable technology’s ambulatory nature, we could measure HRV every 10 seconds throughout the night as participants slept. Previous research, however, required subjects to report to a laboratory or hospital during the day, where experimenters collected HRV measurements for a comparatively brief period (eg, 30 min [29]). In addition to a smaller sampling distribution owing to temporal constraints, the participant’s waking state may have contributed to differences in findings. Time awake has been shown to moderate the effect of the menstrual cycle on HRV ratio, with sleep deprivation significantly increasing sympathetic activity during the midfollicular phase [57]. To better understand why our findings trended in the opposite direction than expected, follow-up studies may wish to directly compare phase-based changes in the HRV ratio across participants’ sleep and waking states.

An additional limitation of our study was that we computed the fertility prediction algorithm’s accuracy based on compliant users who synced their bracelet with the smartphone app at least 80% of the days in a given cycle. Previous research suggests that real-world adherence to reproductive health protocol varies greatly, however. Studies looking at technology-based contraception methods, for example, calculate both the *perfect use* as well as the *typical use* rate of unintended pregnancies, assuming an average user may not follow directions as written all the time (eg, [15,21]). Although we required users to sync their wearable device at least 80% of the days in a given cycle, we attempted to simulate how our algorithm would perform for less compliant users. To do so, we wrote a Python script to randomly remove 10%, 30%, or 50% of the nightly observations from the validation dataset and then recalculated the algorithm’s accuracy and key performance metrics for each simulated amount of noncompliance. As may be expected, the fertility prediction algorithm became increasingly less accurate as more observations were deleted from the dataset. Nevertheless, even with 50% of the observations removed, our fertility algorithm accurately predicted the fertile window in more than 86% of cycles (95% CI 0.85 to 0.89) and had an F1 score equal to 0.72 (95% CI 0.68 to 0.76; see Multimedia Appendix 3 for the performance metrics across all degrees of simulated noncompliance). Planned future analyses will consider the effect of protocol adherence on algorithm prediction accuracy among real-world users. Given our simulated results, we expect a relatively high performance even among users who sync less than 80% of the time.

### Conclusions

Menstrual cycle tracking has numerous applications in health care, further augmented by the development of wearables. First, identification of the fertile window can aid couples planning a pregnancy. A retrospective survey of women who conceived with the help of a fertility monitor found mistiming sexual intercourse to be a leading reason for unexplained infertility; knowledge about their fertile window allowed 49.5% of women in the sample to conceive the subsequent menstrual cycle [58]. Wearables improve upon this possibility, triangulating the fertile window through continuous, high-frequency measurement of multiple parameters. Detection of the fertile window can also assist women who wish to avoid pregnancy but cannot or do not want to use hormonal contraception [15].

Wearable devices provide women with an accurate, convenient alternative to other methods for predicting the fertile window. We have described the measurement, analysis, and interpretation of menstrual cycle–related physiological changes using a wearable device. In our study, wristworn wearables show similar sensitivity and specificity as more invasive, time intensive NFP methods, including cervical mucus monitoring and LH testing [59]. Wearable technology can provide women with real-time insight into their bodies and menstrual cycles, serving as an at-home educational tool. Furthermore, access to cyclic data via a mobile app enables long-term cycle tracking and can lead to more informed lifestyle and medical decisions. Future research should consider how wearable technology can help elucidate physiological patterns underlying women’s health care concerns.

We reproduced the results of previous studies using a more accurate, distributable technology. Significant phase-based differences emerged for 3 of the physiological parameters of interest; the remaining 2 physiological parameters showed similar trends toward phase-based differences, despite a conservative correction to reduce Type I error rates. On the basis of signals collected by the wearable device, we created an algorithm that predicts each woman’s most fertile days in a given cycle. Our study suggests wearables’ imperviousness to confounding factors; the wristworn device detected changes in the menstrual phase over and above daily, cyclical, or individual level fluctuations in parameters. Wearable sensor technology enables the first real-time predictive model of ovulation and represents a valuable addition to women’s health care.

## Appendix

Multimedia Appendix 1 R code output for cross-classified multi-level models of physiological parameters.

Multimedia Appendix 2 Confusion matrix for the Fertility Prediction Algorithm.

Multimedia Appendix 3 Real and simulated Fertility Prediction Algorithm performance for compliant and noncompliant participants.

## Abbreviations

BBT: basal body temperature
BMI: body mass index
bpm: beats per minute
HRV: heart rate variability
LH: luteinizing hormone
NFP: natural family planning
OV: ovulation
WST: wrist skin temperature
