# Supplementary material for: Wearable Sensors Reveal Menses-Driven Changes in Physiology and Enable Prediction of the Fertile Window: Observational Study
Source: J Med Internet Res. 2019 Apr 18;21(4):e13404. doi: 10.2196/13404 (PMC6495289; doi:10.2196/13404)
Supplement: Multimedia Appendix 1 [file jmir_v21i4e13404_app1.docx]

Multimedia Appendix 1. R code output for cross-classified multi-level models of physiological parameters.

## WST

Linear mixed model fit by REML. t-tests use Satterthwaite's method ['lmerModLmerTest']

Formula: temp_skin_percentile_50_cumulative_lowess_filter ~ Phase1 + Phase2 +

Phase3 + Phase4 + (cycle_number | ID) + (Phase1 + Phase2 +

Phase3 + Phase4 | cycle_number)

Data: dt

REML criterion at convergence: 15696.4

Scaled residuals:

Min 1Q Median 3Q Max

-29.9981 -0.5558 0.0134 0.5603 5.7922

Random effects:

Groups Name Variance Std.Dev. Corr

ID (Intercept) 0.718751 0.84779

cycle_number 0.018813 0.13716 -0.52

cycle_number (Intercept) 0.041839 0.20455

Phase1 0.004622 0.06799 -0.55

Phase2 0.009185 0.09584 -0.73 0.88

Phase3 0.006212 0.07881 -0.89 0.75 0.89

Phase4 0.002831 0.05320 -0.19 0.47 0.71 0.41

Residual 0.122790 0.35041

Number of obs: 18761, groups: ID, 193; cycle_number, 16

Fixed effects:

Estimate Std. Error df t value Pr(>|t|)

(Intercept) 34.07995 0.08135 37.82029 418.938 < 2e-16 ***

Phase1 -0.23842 0.02144 5.06475 -11.123 9.46e-05 ***

Phase2 -0.25328 0.02825 4.96390 -8.966 0.000299 ***

Phase3 0.01360 0.02331 3.75087 0.583 0.592987

Phase4 0.19614 0.01790 14.80181 10.955 1.70e-08 ***

---

Signif. codes: 0 ‘***’ 0.001 ‘**’ 0.01 ‘*’ 0.05 ‘.’ 0.1 ‘ ’ 1

Correlation of Fixed Effects:

(Intr) Phase1 Phase2 Phase3

Phase1 -0.334

Phase2 -0.501 0.816

Phase3 -0.607 0.691 0.840

Phase4 - 0.121 0.455 0.672 0.423

## Heart Rate

Linear mixed model fit by REML. t-tests use Satterthwaite's method ['lmerModLmerTest']

Formula: hr_percentile_30_cumulative_lowess_filter ~ Phase1 + Phase2 +

Phase3 + Phase4 + (cycle_number | ID) + (Phase1 + Phase2 + Phase3 + Phase4 | cycle_number)

Data: dt

REML criterion at convergence: 90945.2

Scaled residuals:

Min 1Q Median 3Q Max

-4.4048 -0.6058 -0.0574 0.5364 11.7412

Random effects:

Groups Name Variance Std.Dev. Corr

ID (Intercept) 38.2294 6.1830

cycle_number 0.6467 0.8042 -0.35

cycle_number (Intercept) 0.1733 0.4163

Phase1 0.4729 0.6877 -0.42

Phase2 0.8023 0.8957 -0.77 0.64

Phase3 0.4851 0.6965 -0.87 0.44 0.94

Phase4 1.0929 1.0454 -0.47 -0.18 0.62 0.67

Residual 6.8011 2.6079

Number of obs: 18761, groups: ID, 193; cycle_number, 16

Fixed effects:

Estimate Std. Error df t value Pr(>|t|)

(Intercept) 58.62314 0.44185 181.09444 132.675 < 2e-16 ***

Phase1 -1.54483 0.20179 8.42515 -7.656 4.52e-05 ***

Phase2 -0.03105 0.25575 8.52920 -0.121 0.90619

Phase3 2.00973 0.20241 5.73789 9.929 7.93e-05 ***

Phase4 2.46388 0.28890 6.02276 8.529 0.00014 ***

---

Signif. codes: 0 ‘***’ 0.001 ‘**’ 0.01 ‘*’ 0.05 ‘.’ 0.1 ‘ ’ 1

Correlation of Fixed Effects:

(Intr) Phase1 Phase2 Phase3

Phase1 -0.126

Phase2 -0.204 0.642

Phase3 -0.245 0.451 0.902

Phase4 -0.130 -0.099 0.614 0.650

## HRV

Linear mixed model fit by REML. t-tests use Satterthwaite's method ['lmerModLmerTest']

Formula: HRV_ratio_90 ~ Phase1 + Phase2 + Phase3 + Phase4 + (cycle_number |

ID) + (Phase1 + Phase2 + Phase3 + Phase4 | cycle_number)

Data: dt

REML criterion at convergence: 19793.8

Scaled residuals:

Min 1Q Median 3Q Max

-8.0174 -0.4505 -0.0398 0.4016 15.4466

Random effects:

Groups Name Variance Std.Dev. Corr

ID (Intercept) 0.766123 0.87528

cycle_number 0.015573 0.12479 -0.56

cycle_number (Intercept) 0.106908 0.32697

Phase1 0.013414 0.11582 0.66

Phase2 0.006996 0.08364 -0.88 -0.23

Phase3 0.035019 0.18713 -0.91 -0.74 0.71

Phase4 0.016501 0.12846 -0.83 -0.66 0.62 0.91

Residual 0.153328 0.39157

Number of obs: 18761, groups: ID, 193; cycle_number, 16

Fixed effects:

Estimate Std. Error df t value Pr(>|t|)

(Intercept) 1.70146 0.10259 22.31818 16.585 4.88e-14 ***

Phase1 0.10792 0.03291 4.09896 3.279 0.02944 *

Phase2 0.08433 0.02502 3.48892 3.370 0.03456 *

Phase3 -0.11025 0.04947 5.67101 -2.229 0.06996 .

Phase4 -0.19584 0.03559 4.37562 -5.502 0.00408 **

---

Signif. codes: 0 ‘***’ 0.001 ‘**’ 0.01 ‘*’ 0.05 ‘.’ 0.1 ‘ ’ 1

Correlation of Fixed Effects:

(Intr) Phase1 Phase2 Phase3

Phase1 0.455

Phase2 -0.707 -0.039

Phase3 -0.749 -0.597 0.653

Phase4 -0.662 -0.472 0.570 0.861

## Respiratory Rate

Linear mixed model fit by REML. t-tests use Satterthwaite's method ['lmerModLmerTest']

Formula: br_percentile_90_cumulative_lowess_filter ~ Phase1 + Phase2 +

Phase3 + Phase4 + (cycle_number | ID) + (Phase1 + Phase2 + Phase3 + Phase4 | cycle_number)

Data: dt

REML criterion at convergence: 38343.7

Scaled residuals:

Min 1Q Median 3Q Max

-6.4166 -0.5163 -0.0470 0.4354 17.8265

Random effects:

Groups Name Variance Std.Dev. Corr

ID (Intercept) 3.909348 1.97721

cycle_number 0.037380 0.19334 -0.16

cycle_number (Intercept) 0.002874 0.05361

Phase1 0.014975 0.12237 -0.58

Phase2 0.017698 0.13303 -0.47 0.71

Phase3 0.014329 0.11971 -0.33 0.30 0.80

Phase4 0.008572 0.09259 -0.80 0.39 0.62 0.39

Residual 0.409863 0.64021

Number of obs: 18761, groups: ID, 193; cycle_number, 16

Fixed effects:

Estimate Std. Error df t value Pr(>|t|)

(Intercept) 16.92305 0.14269 194.48072 118.600 < 2e-16 ***

Phase1 -0.39383 0.03953 8.54686 -9.964 5.41e-06 ***

Phase2 -0.48215 0.04161 9.52149 -11.588 6.31e-07 ***

Phase3 -0.20463 0.03765 9.38916 -5.435 0.000357 ***

Phase4 0.21650 0.03211 7.51892 6.743 0.000194 ***

---

Signif. codes: 0 ‘***’ 0.001 ‘**’ 0.01 ‘*’ 0.05 ‘.’ 0.1 ‘ ’ 1

Correlation of Fixed Effects:

(Intr) Phase1 Phase2 Phase3

Phase1 -0.082

Phase2 -0.062 0.674

Phase3 -0.056 0.341 0.759

Phase4 -0.109 0.404 0.597 0.421

## Skin Perfusion

Linear mixed model fit by REML. t-tests use Satterthwaite's method ['lmerModLmerTest']

Formula: perfusion_index_green_percentile_90_cumulative_lowess_filter ~

Phase1 + Phase2 + Phase3 + Phase4 + (cycle_number | ID) + (Phase1 + Phase2 + Phase3 + Phase4 | cycle_number)

Data: dt

REML criterion at convergence: 270025.4

Scaled residuals:

Min 1Q Median 3Q Max

-4.9353 -0.5527 -0.0378 0.5181 5.5482

Random effects:

Groups Name Variance Std.Dev. Corr

ID (Intercept) 447795 669.17

cycle_number 22079 148.59 -0.72

cycle_number (Intercept) 53337 230.95

Phase1 2155 46.42 0.97

Phase2 3320 57.62 0.44 0.41

Phase3 3842 61.98 -0.62 -0.70 0.26

Phase4 9268 96.27 -0.80 -0.83 -0.05 0.93

Residual 95323 308.74

Number of obs: 18761, groups: ID, 193; cycle_number, 16

Fixed effects:

Estimate Std. Error df t value Pr(>|t|)

(Intercept) 1306.538 75.394 22.716 17.329 1.38e-14 ***

Phase1 -44.322 14.796 6.331 -2.995 0.02263 *

Phase2 -73.583 18.248 7.373 -4.032 0.00447 **

Phase3 -12.017 18.486 8.939 -0.650 0.53200

Phase4 51.325 26.955 9.488 1.904 0.08762 .

---

Signif. codes: 0 ‘***’ 0.001 ‘**’ 0.01 ‘*’ 0.05 ‘.’ 0.1 ‘ ’ 1

Correlation of Fixed Effects:

(Intr) Phase1 Phase2 Phase3

Phase1 0.691

Phase2 0.316 0.411

Phase3 -0.443 -0.403 0.370

Phase4 -0.615 -0.561 0.068 0.867
