# Supplementary material for: Wearable Sensors Reveal Menses-Driven Changes in Physiology and Enable Prediction of the Fertile Window: Observational Study
Source: J Med Internet Res. 2019 Apr 18;21(4):e13404. doi: 10.2196/13404 (PMC6495289; doi:10.2196/13404)
Supplement: Multimedia Appendix 2 [file jmir_v21i4e13404_app2.docx]

Multimedia Appendix 2. Confusion matrix for the Fertility Prediction Algorithm

$$\left[ \begin{matrix} \boldsymbol{752} & 74 & 0 \\ 41 & \boldsymbol{413} & 56 \\ 0 & 58 & \boldsymbol{1000} \end{matrix} \right]$$

*Note:* Each row of the confusion matrix corresponds to the predicted class. In descending order, these are: follicular phase, fertile window, and the luteal phase. Each column corresponds to the actual class of the observed cycle day. The bolded matrix diagonal indicates the number of true positives for each class.
