# Supplementary material for: Wearable Sensors Reveal Menses-Driven Changes in Physiology and Enable Prediction of the Fertile Window: Observational Study
Source: J Med Internet Res. 2019 Apr 18;21(4):e13404. doi: 10.2196/13404 (PMC6495289; doi:10.2196/13404)
Supplement: Multimedia Appendix 3 [file jmir_v21i4e13404_app3.docx]

Multimedia Appendix 3. Real and simulated Fertility Prediction Algorithm performance for compliant and noncompliant participants

|  | Accuracy  (95% CI) | Sensitivity/Recall (95% CI) | Specificity  (95% CI) | Precision  (95% CI) | F score_means_ (95% CI) |
| --- | --- | --- | --- | --- | --- |
|  |  |  |  |  |  |
| Adherent Participants  ($\boldsymbol{\geq80\%}$ cycle days synced) |  |  |  |  |  |
|  | 0.9048 (0.8891, 0.9205) | 0.8098 (0.7680, 0.8516) | 0.9303 (0.9189, 0.9417) | 0.7650 (0.7262, 0.8039) | 0.7812  (0.7432, 0.8192) |
| Simulated Non-Adherence |  |  |  |  |  |
| 10% of cycle days removed | 0.8982 (0.8812, 0.9151) | 0.7941 (0.7514, 0.8368) | 0.9256 (0.9131, 0.9381) | 0.7514 (0.7093, 0.7934) | 0.7765 (0.7400, 0.8129) |
| 30% of cycle days removed | 0.8759 (0.8560, 0.8958) | 0.7471 (0.6998, 0.7943) | 0.9103 (0.8965, 0.9242) | 0.6988 (0.6535, 0.7441) | 0.7278 (0.6859, 0.7698) |
| 50% of cycle days removed | 0.8694 (0.8501, 0.8888) | 0.7412 (0.6969, 0.7855) | 0.9040 (0.8897, 0.9182) | 0.6865 (0.6428, 0.7301) | 0.7182 (0.6785, 0.7579) |
